# Validation of a continuous low-dose iohexol infusion to measure the glomerular filtration rate

**DOI:** 10.1186/cc10965

**Published:** 2012-03-20

**Authors:** J Dixon, K Lane, N Dalton, I MacPhee, B Philips

**Affiliations:** 1 St George's Hospital, London, UK; 2 King's College, London, UK

## Introduction

We have designed a method of continuous measurement of the glomerular filtration rate (GFR) with the intention of applying the method in patients with acute kidney injury (AKI). The aim of the study was to prove the method in healthy volunteers (HV) and patients with chronic kidney disease (CKD).

## Methods

HV and patients with CKD were randomly allocated to measurement of GFR using iohexol, either by the established method of single injection and measurement of its rate of elimination (gold standard), or by the continuous infusion of a very low dose of iohexol (0.5 ml/hour for 12 hours). The GFR was measured again, using the other method, after a washout period of 4 to 28 days. Plasma iohexol concentration was measured at 10 time points and plotted on a two-phase exponential decay graph. The GFR was calculated by dividing the infusion concentration by the plateau concentration. The *t *test compared results with 4-hour creatinine clearance (4-CrCl), and the CKD-EPI equation.

## Results

Six HV and seven CKD patients volunteered, with five in each group completing the study. There was no difference between the two groups (*P *= 0.79). In HV, the mean GFR measured by single injection was 105 ± 7.3 and 109.4 ± 9.9 ml/minute/1.73 m^2 ^by infusion (Pearson *r *= 0.95, *P *= 0.0002). In CKD patients, the mean GFR measured by single injection was 40 ± 5.4 and 44.8 ± 6.2 ml/minute/1.73 m^2 ^by infusion (Pearson *r *= 0.99, *P *< 0.0001). The infusion method depends on reaching a steady plasma concentration, which took 165 ± 84 minutes in HV and 483 ± 127 minutes in CKD patients to be within 10% of the steady state. The GFR is overestimated by 4-CrCl (by 13.9 ± 12.9 ml/minute/1.73 m^2^, *P *< 0.0001) and by CKD-EPI (by 8.4 ± 9.6 ml/minute/1.73 m^2^, *P *< 0.0001).

## Conclusion

In future work, we aim to validate this method in critically ill patients with AKI. We predict the steady state achieved will be increased. Anticipated problems include increased time or failure to reach steady state. However, given the simplicity of the method we hypothesise that changes in iohexol concentration may provide valuable real-time information about the GFR in AKI. Changes are likely to occur before serum creatinine rises. In conclusion, the continuous iohexol infusion method of measuring GFR appears to be accurate and precise. In stable subjects, a steady plasma concentration is achieved before it is observed with creatinine changes.

